# Is Timing of Enrollment Associated with Birth Outcomes? Findings from a Healthy Start Program in Kansas

**DOI:** 10.1007/s10995-017-2405-x

**Published:** 2017-11-28

**Authors:** Kyrah K. Brown, Candace Johnson, Michele Spainhower, Nicole Fox Phillips, J’Vonnah Maryman

**Affiliations:** 1 Division of Public Health Performance, Sedgwick County Health Department, 1900 E. 9th Street, Wichita, KS 67206 USA; 2 Division of Children and Family Health, Sedgwick County Health Department, 1900 E. 9th Street, Wichita, KS 67206 USA

**Keywords:** Preterm birth, Low birth weight, Race/ethnicity, Healthy start, Life course

## Abstract

*Objective* The Sedgwick County Healthy Babies Healthy Start (HBHS) program provides community-based services (including care coordination, interconception care and home visiting) aimed at reducing racial/ethnic disparities in poor birth outcomes. The purpose of this study is to assess the effectiveness of the Sedgwick County HBHS program by comparing the birth outcomes of program participants who enrolled prenatally and those who did not participate while pregnant. *Methods* In this retrospective cohort study, we used data drawn from the Sedgwick County HBHS program. The sample included 280 clients who were enrolled in the Sedgwick County HBHS program between September 2014 and December 2015. We performed logistic regression analyses to assess the associations between enrollee type (prenatal enrollee vs. interconceptional enrollee) and birth outcomes (low birth weight, preterm birth). *Results* The majority of the sample consisted of racial/ethnic minority women (32.1% non-Hispanic black, 31.8% Hispanic). After adjusting for covariates, women who enrolled in the Sedgwick County HBHS program prenatally were less likely than women who were not enrolled during pregnancy to have a preterm birth (OR 0.19, [CI 08, 0.43]) and deliver a low birth weight infant (OR 0.31, [CI 0.10, 0.97]). *Conclusions for Practice* Women, particularly minority women, who participate in the HBHS program experienced better birth outcomes than women who did not participate in the program during pregnancy. However, findings also suggest that interconceptional enrollees may stand to benefit from continued participation in the program.

## Significance Statement

Healthy Start has three distinct program features (care coordination, interconception care, and home visiting) which have been shown to improve maternal and infant health outcomes. Our findings reveal that women who participated in the Sedgwick County Healthy Babies Healthy Start program had better birth outcomes than women who did not participate during their pregnancy. These findings have important implications for future initiatives focused on minority women.

## Background

In the United States, preterm birth (birth at >37 weeks gestation) and low birth weight (weighing >2500 g at birth) are leading causes of infant mortality and morbidity (Mathews and MacDorman 2013). Among infants who survive preterm birth (PTB) or low birth weight (LBW), there is an increased risk of acute complications (e.g., respiratory distress syndrome, interventricular hemorrhage, infections) and long-term physical and neurodevelopmental disabilities (e.g., cerebral palsy, intestinal problems, vision or hearing loss, attention deficit-hyperactive disorder) (Barre et al. 2011; Boulet et al. 2011; Butler and Behrman 2007). Despite an 11% decline in the PTB rate and a relatively stable LBW rate, significant socioeconomic and racial and ethnic disparities persist. For example, non-Hispanic Black women continue to have disproportionately higher PTB/LBW rates compared to non-Hispanics Whites (Martin et al. 2015).

Disparities in birth outcomes are believed to be a result of differential exposures to biological, psychological and social events during pregnancy and over the lifespan (Halfon and Hochstein 2002; Lu et al. 2010; Lu and Halfon 2003). Previous research suggests the period of time before and after pregnancy serve as critical windows of opportunity to intervene and optimize women’s health. Therefore, comprehensive approaches that incorporate multilevel intervention strategies, cross-sector collaboration (i.e., service coordination and integration to holistically address social determinants of health) and multidimensional systems integration across the life course are key in reducing disparities in birth outcomes (Institute of Medicine 2002; Lu 2014; Lu et al. 2010; Williams 2012).

## National Healthy Start Initiative

The National Healthy Start Initiative (hereafter referred to as Healthy Start), initiated in 1991, is the largest multilevel, federally-sponsored program to address racial and ethnic disparities in birth outcomes (Rosenbach et al. 2010). Healthy Start has three distinct features: maternal care coordination, interconception care and home visiting. Maternal care coordination includes prenatal case management by a health or social service professional. Previous research has documented the effectiveness of maternal care coordination in increasing service utilization (Buescher et al. 1991; Murfet et al. 2014; Piper et al. 1996), increasing early initiation of prenatal care (Cramer et al. 2007), and improving maternal health status or behaviors (Hillemeier et al. 2014; Margolis et al. 2001). Although several studies have reported improvements in LBW (Kothari et al. 2014; Rosenbach et al. 2010; Salihu et al. 2008), PTB (Salihu et al. 2008) and infant death (Levandowski et al. 2006), the evidence remains mixed (Cramer et al. 2007; Rosenbach et al. 2010; Smith et al. 2010).

Interconception care, or care that is provided between pregnancies (Rosenbach et al. 2010) has been shown to improve women’s health behaviors (e.g., physical activity, dental visit, contraceptive use), knowledge and attitudes regarding preconception health (Coonrod et al. 2014). Participation in interconception care intervention has also been associated with increased identification and treatment of untreated chronic diseases and conditions, increased reproductive and contraceptive planning, and increased interpregnancy intervals (Biermann et al. 2006). The success of home visiting services in improving maternal health outcomes is also well documented. This includes overall health status (e.g., pregnancy-induced hypertension) (Filene et al. 2013; Kitzman et al. 1997), health-promoting behaviors (e.g., breastfeeding) (Olds et al. 1998, 2010; Shah and Austin 2014), access to and use of services (Issel et al. 2011), and improved skills related to infant caregiving (Filene et al. 2013; Kitzman et al. 1997; Olds et al. 1998).

### Sedgwick County Healthy Babies Healthy Start

Sedgwick County has the largest proportion of infant deaths in Kansas with approximately 24% of those deaths in the county caused by prematurity/LBW (Kansas Department of Health and Environment 2015). Moreover, prematurity/LBW contributed to 33.1% of infant deaths among non-Hispanic Blacks, 23.3% among Hispanics and 15.1% among non-Hispanic Whites (Kansas Department of Health and Environment 2015). Initiated in 1997, the Sedgwick County Health Department Healthy Babies Healthy Start (HBHS) program uses the Healthy Start framework to address health needs and reduce disparities in adverse birth outcomes. The program targets high-risk women residing in eight zip codes with high rates of infant mortality. Among all of the women served by Sedgwick County HBHS, 48% are enrolled interconceptionally (enrolled within 3 months of most recent birth) and are usually referred to the program by health care providers. Sedgwick County HBHS’s newly implemented program model provides multilevel services, including maternal care coordination, interconception care and home visiting.

The purpose of this study is to assess the effectiveness of the Sedgwick County HBHS program by comparing the birth outcomes of program participants who enrolled prenatally and those who did not participate while pregnant.

## Methods

### Data Source and Sample

The data for this study was obtained from the de-identified electronic health records (EHR) of Sedgwick County HBHS program participants. Program staff exported participant data from their EHR database into a spreadsheet that included the data records of 433 women who were enrolled during the first phase of the Healthy Start grant year (September 2014–December 2015). Records for participants who were currently pregnant or had not delivered an infant during the 15-month time period were excluded. The resulting analytic sample included 280 clients. Two-hundred and twenty-two clients were ‘prenatal enrollees’ who enrolled in the program while pregnant and participated in the program a minimum of 3 months prior to delivery. Fifty-six clients were ‘interconceptional enrollees’ who enrolled in the program within 3 months after a delivery. During intake, the Sedgwick County HBHS medical providers complete interconceptional enrollment and assessment forms with interconceptional enrollees. The interconceptional enrollment and assessment forms were designed by the Sedgwick County HBHS EHR provider in conjunction with HRSA to ensure the collection of data necessary for HRSA grant reporting requirements.

This study was reviewed and approved by the Institutional Review Board at the Kansas Department of Health and Environment.

### Measures

The primary outcome measures were low birth weight (LBW) and preterm birth (PTB). LBW was measured with a dichotomous variable indicating whether birthweight was less than 2500 g (1 = yes, 0 = no). PTB was also measured with a dichotomous variable indicating whether a birth occurred at less than 37 weeks gestation (1 = yes, 0 = no). The predictor variable was a dichotomous variable representing timing of enrollment (1 = prenatally enrolled, 0 = interconceptionally enrolled).

Covariates included employment status (1 = employed, 2 = unemployed, 3 = other), insurance status (1 = insured, 0 = uninsured), and chronic health condition (1 = presence of a chronic health condition present, 0 = absence of a chronic health condition). Other demographic characteristics included age (1 = under 19 years old, 2 = 20–34 years old, 3 = 35 and older), race/ethnicity (1 = Non-Hispanic white, 2 = Non-Hispanic black, 3 = Hispanic), primary language spoken (1 = English, 2 = Spanish, 3 = Other), marital status (1 = single, 2 = married, 3 = divorced/separated), and highest level of education completed (1 = High School diploma, 0 = No High School diploma).

### Statistical Analyses

Percentages were calculated to summarize the demographic characteristics of women included in the present study. Chi square analyses were conducted to evaluate potential differences between women who were enrolled in the HBHS program prenatally and women who were not (interconceptional enrollees). Demographic variables that were statistically significant were included as covariates in the logistic regression models. Two logistic regressions were conducted to assess associations between timing of enrollment (prenatally enrolled or interconceptionally enrolled), preterm birth and low birth weight. Covariates included in the regression models were employment status, insurance status and chronic health condition. All statistical tests were two-tailed with significance accepted at the *p* < .05 level. Statistical analyses were conducted using SPPS Version 21.

## Results

As shown in Table 1, one-third of all HBHS clients were either non-Hispanic black (N = 90, 32.1%) or Hispanic (N = 89, 31.8%). The majority of women were between 20 and 34 years old (N = 185, 66.1%), primarily English speakers (N = 225, 80.4%), single, never married (N = 201, 71.8%) and did not have a high school diploma.

**Table 1:** Demographic characteristics by enrollee type, Sedgwick County HBHS Program

Variables	Prenatal enrollees (n = 224)	Interconception enrollees (n = 56)	Total (n = 280)	P value
Age				
Under 19 years	23(11.5%)	3 (6%)	26 (9.3%)	*p* = .521
20–34 years	146 (73%)	39 (78%)	185 (66.1%)	
35 and older	31(15.5%)	8 (16%)	39 (13.9%)	
Missing	24 (10.7%)	6 (10.7%)	30 (10.7)	
Race/ethnicity				
NH white	54 (24.1%)	20 (35.7%)	74 (26.4%)	*p* = .35
NH black	74 (33%)	16 (28.6%)	90 (32.1%)	
Hispanic	73 (32.6%)	16 (28.6%)	89 (31.8%)	
Other racial groups	23 (10.3%)	4 (7.1%)	27 (9.6%)	
Primary language spoken				
English	117 (79%)	48 (85.7%)	225 (80.4%)	*p* = .42
Spanish	44 (19.6%)	8 (14.3%)	52 (18.6%)	
Other	3 (1.3%)	–	3 (1.1%)	
Marital status				
Single	158 (70.5%)	43 (76.8%)	201 (71.8%)	*p* = .35
Married	55 (24.6%)	9 (16.1%)	64 (22.9%)	
Divorced/separated	11 (24.6%)	4 (7.1%)	15 (5.4%)	
Education completed				
Not HS graduate	86 (40.6%)	21 (43.8%)	107 (38.2%)	*p* = .68
HS graduate	126 (59.4%)	27 (56.3%)	153 (54.6%)	
Missing	12 (5.3%)	8 (14.2%)	20 (7.1%)	
Employment status				
Employed	67 (29.9%)	8 (14.3%)	75 (26.8%)	***p*** < .**01**
Unemployed	106 (47.3%)	40 (71.6%)	146 (52.1%)	
Student	32 (14.3%)	2 (3.6%)	34 (12.1%)	
Missing	19 (8.4%)	6 (10.7%)	25 (8.9%)	
Insurance status				
Insured	170 (75.9%)	38 (67.9%)	208 (74.3%)	*p* = .21
Uninsured	54 (24.1%)	18 (32.1%)	72 (25.7%)	
Chronic health condition				
Yes	110 (50%)	36 (67.9%)	146 (52.1%)	***p*** < .**05**
No	110 (50%)	17 (32.1%)	127 (45.4%)	
Missing	4 (1.7%)	3 (5.3%)	7 (2.5%)	

Bold means that the test result was statistically significant

As shown in Table 1, prenatal enrollees were demographically similar to women who were not enrolled during their pregnancy in terms of age, race/ethnicity, marital status and highest level of education completed. Women who were enrolled in the program prenatally were less likely than those who did not enroll prenatally to be unemployed and to have a chronic health condition.

Table 2 shows the regression coefficients, standard error, Wald’s statistic, odds ratios, 95% confidence intervals for odds ratios, and Hosmer–Lemeshow tests for two regression models.

**Table 2:** Logistic regression results examining differences in birth outcomes among Sedgwick County HBHS prenatal enrollees compared to women who were not enrolled prenatally

	Preterm delivery					Low birthweight delivery				
Variable	B	SE	Wald X^2^	DF	OR (C.I.)^a^	B	SE	Wald X^2^	DF	OR (C.I.)^a^
Predictor variable										
Prenatal enrollees^b^	− 1.65	0.42	0.19	1	0.19 (0.08, 0.43)**	− 1.14	0.57	4.01	1	0.31 (0.104, 0.975)*
Covariates										
Employed^c^	− 0.50	0.67	0.56	1	0.61 (0.16, 2.25)	0.38	0.76	0.26	1	1.47 (0.33, 6.54)
Unemployed^d^	− 0.59	0.59	1.01	1	0.55 (0.17, 1.75)	− 0.06	0.71	0.01	1	0.94 (0.23, 3.78)
Chronic health condition present^e^	− 0.34	0.40	0.73	1	0.71 (0.31, 1.56)	0.30	0.52	0.32	1	1.35 (0.48, 3.80)
Hosmer & lemeshow test		Test statistic = 1.13 df = 7, p = .99 88.6%					Test statistic = 2.71 df = 6, p = .84 94.1%			
Predictive accuracy										

^a^Odds ratio (confidence interval). reference group = interconceptional enrollees

^b^Reference group: interconceptional enrollees

^c^Reference group: unemployed

^d^Reference group: other

^e^Reference group: absence of chronic health condition. (*p < .05, **p < .01)

Logistic regression results indicated that after adjusting for employment status and chronic health conditions, women who were enrolled in the HBHS program prenatally were five times less likely than women who were not enrolled in the HBHS program during pregnancy to deliver preterm (OR 0.19, [CI 0.08, 0.43]). The regression model demonstrated high predictive accuracy (88.6%) and good fit, *t*(7) = 1.13, p = .99.

After adjusting for employment status and chronic health conditions, women who were enrolled in the HBHS program prenatally were also three times less likely than women who were not enrolled in the program during pregnancy to deliver a low birthweight infant (OR 0.31, [CI 0.10, 0.97]). The regression model demonstrated high predictive accuracy (94.1%) and good fit, *t*(6) = 2.71, p = .84.

## Discussion

The findings from this study suggest that the provision of maternal care coordination and home visitation in an integrated model during pregnancy may favorably impact women’s birth outcomes. The majority of the women participating in the Sedgwick County HBHS program were non-Hispanic black and Hispanic. Considering that babies being born preterm/LBW account for 33.1% of infant deaths among non-Hispanic blacks and 23.3% of infant deaths among Hispanics(Kansas Department of Health and Environment 2015), the Sedgwick County HBHS program demonstrates the potential to mitigate these statistics. The effects of the program are seen even after controlling for social determinants of health such as employment and chronic health conditions.

### Limitations

Several limitations should be considered in interpreting the study findings. First, there are limitations regarding the comparison of the two groups. In the interest of understanding differences in outcomes based on timing of enrollment, we compared the birth outcomes of prenatal enrollees and interconceptional enrollees. However, birth outcomes for interconceptional enrollees were based on retrospective self-reported information gathered by a medical provider during program intake. In many cases, the Sedgwick County HBHS program is not able to obtain all records for interconceptional enrollees. In contrast, outcomes for prenatal enrollees were gathered from records obtained while the client was enrolled in the Sedgwick County HBHS program. It is possible that interconceptional women, who enrolled in Sedgwick County HBHS after a recent birth, may have been subject to recall bias. Second, prenatal enrollees were overrepresented in the study sample which could have influenced the findings. Third, the small sample size limits our ability to generalize findings to a larger population of Healthy Start participants. Finally, HBHS provides a set of multifaceted interventions, and it was not possible to tease apart which specific intervention contributed to the study’s findings. Although we did not focus on process, we attempted to provide some insight into program delivery characteristics using available data.

### Implications for Practice and Future Research

Despite study limitations, our findings contribute to the empirical literature on the benefits of enrollment in HBHS programs using an integrated model on birth outcomes among racially and ethnically diverse clients. High-risk racial/ethnic minority women, in particular, stand to benefit from this program. This has specific implications for Sedgwick County, KS which is challenged by high preterm birth rates. The present study also offers important insights into the interconception client population who may enter into the program with more health risks and less support. It is important to recognize that interconceptional clients may stand to benefit from long-term participation in the HBHS program. For example, by providing interconception care services to women in a non-Medicaid expansion state, HBHS is able to fill important gaps in access. Future longitudinal studies are needed that follow women over time to study temporality between HBHS participation and birth outcomes. Future studies should also consider employing rigorous methods to assess HBHS program dosage and intended program outcomes. Moreover, future studies should not only consider examining the effects of each program component on clients’ health and birth outcomes, but also how they may differentially impact outcomes among program participants. Future program design can be enhanced by research identifying the most important aspects of the integrated model that contribute to favorable outcomes.
